# Complete Tubeless video-assisted thoracoscopic surgery for pulmonary nodules: association with faster recovery, less pain, and shorter hospital stay

**DOI:** 10.3389/fsurg.2026.1864331

**Published:** 2026-08-19

**Authors:** Zhenlin Jiang, Jianhua Yan, Yuanxiang Shi, Jianjun Jiang, Jinsong Yang, Zuxin Dong, Junting Chen

**Affiliations:** 1 Department of Thoracic Surgery, Hunan Provincial People’s Hospital (The First Affiliated Hospital of Hunan Normal University), Changsha, Hunan, China; 2 Central Laboratory, Hunan Provincial People's Hospital (The First Affiliated Hospital of Hunan Normal University), Changsha, Hunan, China; 3 Department of Thoracic Surgery, Changsha Fourth Hospital, Changsha, Hunan, China

**Keywords:** faster recovery, pulmonary nodule, spontaneous respiration, Tubeless, video-assisted thoracoscopy

## Abstract

**Background:**

Conventional video-assisted thoracoscopic surgery (VATS) with intubation and chest drainage is associated with increased postoperative pain and delayed recovery. This retrospective, non-randomized comparative cohort study evaluated the safety and feasibility of a complete Tubeless VATS approach (spontaneous breathing with laryngeal mask anesthesia, without urinary catheter or chest tube) for peripheral pulmonary nodules in young and middle-aged patients.

**Methods:**

Between September 2022 and December 2023, 50 patients undergoing wedge or segmental resection for pulmonary nodules (≤3 cm) were enrolled. Twenty-five received Tubeless VATS (experimental group) and 25 received conventional VATS (control group). Perioperative outcomes included operative time, blood loss, time to first oral intake, hospital stay, anesthesia recovery time, visual analog scale (VAS) pain scores, PetCO_2_, SaO_2_, postoperative day 1 inflammatory markers [white blood cell count, neutrophil-to-lymphocyte ratio (NLR)], complications, and MD Anderson symptom inventory (MDASI) patient-reported outcomes (postoperative days 1–7 and weeks 2–4).

**Results:**

All 50 surgeries were successful without conversion to open surgery or endotracheal intubation. Compared with conventional VATS, Tubeless VATS was associated with significantly shorter time to first oral intake (5.12 ± 0.83 vs. 7.68 ± 1.11 h), shorter hospital stay (3.00 ± 1.04 vs. 4.84 ± 0.89 days), faster anesthesia recovery (11.12 ± 5.74 vs. 23.80 ± 5.82 min), lower VAS pain scores at all postoperative time points (*P* < 0.01), lower postoperative white blood cell count (10.67 ± 1.13 vs. 11.75 ± 1.62 × 10⁹/L), lower NLR (8.23 ± 2.48 vs. 9.85 ± 3.12), and fewer complications (8% vs. 32%, *P* = 0.034), with all conventional group complications being Grade I and predominantly related to intubation and catheterization. MDASI scores were better on days 1–5 (*P* < 0.05) but not thereafter. Maximum intraoperative PetCO_2_ was higher in the Tubeless group (51.20 ± 4.81 vs. 42.64 ± 2.99 mmHg, *P* = 0.023), with no significant difference in postoperative PaCO_2_ (44.08 ± 3.13 vs. 42.28 ± 3.97 mmHg, *P* = 0.082). Operative time and blood loss did not differ significantly between groups.

**Conclusion:**

In selected young and middle-aged patients with peripheral pulmonary nodules ≤3 cm undergoing wedge or segmental resection, complete Tubeless VATS appears safe and feasible. It was associated with reduced postoperative pain, shorter hospital stay, lower inflammatory response, fewer complications, and improved early patient-reported outcomes, consistent with enhanced recovery after surgery principles. This Tubeless technique may represent a surgical innovation worthy of further evaluation in clinical practice.

## 1 Introduction

A pulmonary nodule is defined as a round or round-like lesion within the lung parenchyma with a diameter of ≤3 cm. With the widespread use of high-resolution computed tomography, the current detection rates of pulmonary nodules have reached 22%–51%, of which approximately 10% harbor malignant potential (1). For nodules highly suspected of being early-stage non-small cell lung cancer (NSCLC), surgical intervention remains the gold standard for both diagnosis and treatment (2). Accumulating evidence demonstrates that sublobar resections, including wedge resection and anatomical segmentectomy, achieve 5-year survival rates comparable to lobectomy for small-sized peripheral NSCLC, while preserving more lung function and reducing morbidity (3). Concurrently, video-assisted thoracoscopic surgery (VATS) has largely replaced thoracotomy due to its superior visualization, reduced trauma, and faster recovery (4).

The concept of enhanced recovery after surgery (ERAS) aims to shorten hospital stay, improve rehabilitation quality, and optimize medical resources (5). An innovative extension of ERAS in thoracic surgery is the “Tubeless” approach, which preserves spontaneous breathing without endotracheal intubation, avoids muscle relaxants, omits urinary catheters, and most distinctively, omits postoperative chest drainage (6). By preserving spontaneous breathing, Tubeless anesthesia reduces anesthetic drug exposure, decreases postoperative nausea and vomiting, and facilitates earlier return of gastrointestinal function (7). Furthermore, avoiding a chest tube significantly reduces postoperative pain, promotes early ambulation, and shortens hospital stay.

The feasibility of awake wedge resection under epidural anesthesia was first demonstrated by Pompeo et al. (8). Subsequently, non-intubated anesthesia was extended to more complex procedures, including uniportal VATS lobectomy with systematic lymph node dissection (9). The safety and feasibility of non-intubated VATS for various thoracic diseases have been further supported by a large meta-analysis encompassing 1,684 cases (10). Spontaneous ventilation Tubeless thoracoscopic sympathectomy has been successfully applied in 144 patients with primary palmar hyperhidrosis, achieving an average hospital stay of only 1 day (11). For primary spontaneous pneumothorax, non-intubated VATS resulted in shorter anesthesia recovery time, shorter postoperative hospital stay, and lower pain scores compared with conventional intubated VATS (12). In addition, Tubeless VATS has been successfully performed in 67 patients with mediastinal tumors, with zero postoperative complications reported at discharge (13).

Despite these promising findings, several research gaps persist. First, most studies have focused on non-intubated anesthesia but have not fully implemented the complete Tubeless concept—the simultaneous avoidance of endotracheal tube, urinary catheter, and chest tube. Second, published series have included heterogeneous populations with wide age ranges and varying comorbidities, limiting the ability to assess safety, specifically in young and middle-aged patients with low perioperative risk. Third, previous studies have rarely incorporated patient-reported outcomes (PROs) or serial postoperative inflammatory markers such as neutrophil-to-lymphocyte ratio (NLR), platelet-to-lymphocyte ratio (PLR), and systemic immune-inflammation index (SII), which are sensitive indicators of surgical stress and recovery quality. Fourth, no study has systematically compared Tubeless versus conventional VATS using both PROs and objective inflammatory biomarkers in a well-defined cohort of young patients undergoing wedge or segmental resections for pulmonary nodules.

Therefore, the present study was designed to address these gaps. We aimed to evaluate the safety and feasibility of complete Tubeless VATS compared with conventional intubated VATS in young and middle-aged patients (20–55 years) with American Society of Anesthesiologists (ASA) class ≤2 and body mass index (BMI) ≤30 kg/m^2^ undergoing wedge or segmental resection for peripheral pulmonary nodules ≤3 cm. We collected and compared perioperative indicators, anesthesia parameters, visual analog scale (VAS) pain scores, complication rates, longitudinal PROs [MD Anderson symptom inventory (MDASI) scores from postoperative day (POD) 1 through week 4], and early inflammatory markers. We hypothesized that Tubeless VATS would be associated with faster recovery, less postoperative pain, reduced inflammatory response, fewer complications, and shorter hospital stays, providing preliminary evidence to support this procedure as a viable alternative for selected young and middle-aged patients with pulmonary nodules in alignment with ERAS principles.

## 2 Methods

### 2.1 Study design and ethical approval

This was a single-center, retrospective comparative cohort study conducted in accordance with the principles of the Declaration of Helsinki. The protocol was approved by the Ethics Committee of Hunan Provincial People's Hospital, Changsha, Hunan, China (Approval Number: 2022-82). All patients provided written informed consent for their surgical procedure and for the use of their anonymized data for research purposes.

### 2.2 Study subjects

Consecutive patients with pulmonary nodules who underwent Tubeless VATS or conventional VATS at the Department of Thoracic Surgery, Hunan Provincial People’s Hospital between September 2022 and December 2023 were enrolled.

#### 2.2.1 Inclusion criteria

(1) Age 20–55 years; (2) BMI ≤ 30 kg/m^2^; (3) ASA class ≤2; (4) no significant cardiopulmonary dysfunction; (5) no history of thoracic surgery or recent respiratory tract infection; and (6) chest CT showing a peripheral pulmonary nodule ≤3 cm in maximum diameter, deemed to indicate surgical intervention after multidisciplinary team discussion.

#### 2.2.2 Exclusion criteria

(1) Coagulation dysfunction; (2) hemodynamic instability; (3) difficult intubation (Mallampati grade ≥3); (4) preoperative hypoxemia (PaO_2_ < 60 mmHg) and/or hypercapnia (PaCO_2_ > 50 mmHg); and (5) intraoperative finding of extensive pleural adhesions.

### 2.3 Group assignment

Patients were allocated to either the Tubeless group or the conventional VATS group based on the anesthesia method selected by the surgical and anesthesia team. Allocation was not randomized; rather, patients were consecutively enrolled and assigned based on clinical decision-making and patient preference. All patients were informed preoperatively about the surgical plan and risks and provided signed informed consent.

### 2.4 Anesthesia methods

#### 2.4.1 Experimental group (Tubeless VATS)

Patients received general anesthesia with a laryngeal mask combined with multimodal nerve blocks: ultrasound-guided single-point injection of 0.375% ropivacaine 20–25 mL at the T3–T5 level for paravertebral block; 2% lidocaine for incision site local infiltration; a 1:1 mixture of 0.75% ropivacaine and 2% lidocaine for intercostal nerve block; spraying 10 mL of 2% lidocaine on the pulmonary hilum and visceral pleura for pleural surface anesthesia; and 2% lidocaine for vagus nerve block. Spontaneous breathing was preserved throughout. Conversion to endotracheal intubation was performed if SaO_2_ fell below 90% despite assisted ventilation with increased FiO_2_ and jaw-thrust maneuver, or if hemodynamic instability (systolic blood pressure <90 mmHg) occurred despite fluid resuscitation. No urinary catheter was placed. No chest drainage tube was inserted postoperatively unless clinically indicated.

#### 2.4.2 Control group (conventional VATS)

Patients received conventional general anesthesia with double-lumen endotracheal tube intubation and mechanical one-lung ventilation. Urinary catheters were placed intraoperatively. A chest drainage tube was inserted postoperatively and removed when drainage was <200 mL/24 h, no air leak was present upon coughing, and chest X-ray confirmed good lung expansion.

### 2.5 Surgical methods

All surgeries were performed by the same surgical team. For wedge resection, the lesion was located preoperatively using three-dimensional CT reconstruction or intraoperatively by palpation. A margin of ≥2 cm was ensured, and the nodule was completely resected with a linear stapler and sent for frozen section examination. For segmentectomy, the segmental artery, vein, and bronchus were identified after preoperative three-dimensional localization, and the target segment was resected with a linear stapler. If frozen section suggested malignancy, sampling of mediastinal lymph node stations 7, 10, and 11 was performed. If lymph nodes were positive on frozen section, lobectomy was performed. If the frozen section was benign, the chest was closed.

### 2.6 Postoperative management

After returning to the cardiothoracic surgery intensive care unit, patients underwent routine monitoring of vital signs. A bedside chest X-ray was performed on the day of surgery. Patients started drinking water 4–6 h postoperatively and progressed to a normal diet if tolerated. Patients with stable conditions were transferred to the general ward on POD 1. Urinary catheters in the control group were removed on POD 1. Patients were monitored for complications, including urinary retention, hoarseness, nausea, vomiting, and choking.

Postoperative analgesia was standardized for both groups: Patients were intravenously administered tramadol 50 mg as rescue analgesia when VAS score exceeded 4, and ondansetron 4 mg was administered intravenously as needed for nausea or vomiting. Mobilization was encouraged within 6–8 h postoperatively, and patients were ambulated with assistance as tolerated. Discharge criteria included tolerating oral intake, ambulating independently, VAS score ≤3, and no active complications. In the control group, the chest tube was removed when drainage was <200 mL/24 h, no air leak was present upon coughing, and chest X-ray confirmed good lung expansion.

### 2.7 Data collection

#### 2.7.1 Perioperative clinical indicators

General patient information was collected, including smoking history, nodule size, pathological type, surgical method, operation time, intraoperative blood loss, time to first postoperative oral intake, postoperative complications, postoperative hospital stay, and PROs. Postoperative inflammatory markers measured on POD 1 included white blood cell count, platelet count, neutrophil count, lymphocyte count, NLR, PLR, and SII. Pain was assessed using the VAS (0–10). PROs were assessed using the MDASI, selecting items for pain, fatigue, sleep, breathing, cough, appetite, and constipation (each scored 0–10).

#### 2.7.2 Anesthesia-related indicators

Recorded anesthesia parameters included maximum intraoperative end-tidal carbon dioxide partial pressure (PetCO_2_), mean intraoperative PetCO_2_, minimum intraoperative oxygen saturation (SaO_2_), anesthesia induction time, anesthesia recovery time, and postoperative PaCO_2_ upon return to the ward.

### 2.8 Statistical analysis

Statistical analyses were performed using SPSS version 26.0. Normality was tested using the Shapiro–Wilk test. Normally distributed continuous data were expressed as mean ± standard deviation and compared using independent samples *t*-tests. Non-normally distributed data were compared using the Wilcoxon rank-sum test. Categorical variables were expressed as frequencies and percentages, and compared using the chi-square test or Fisher's exact test as appropriate. Given the exploratory nature of this study, no adjustment for multiple comparisons was applied. No *a priori* sample size calculation was performed, as this was a retrospective feasibility study. A two-tailed *P*-value <0.05 was considered statistically significant.

## 3 Results

### 3.1 Patient disposition and baseline characteristics

**Figure 1:**
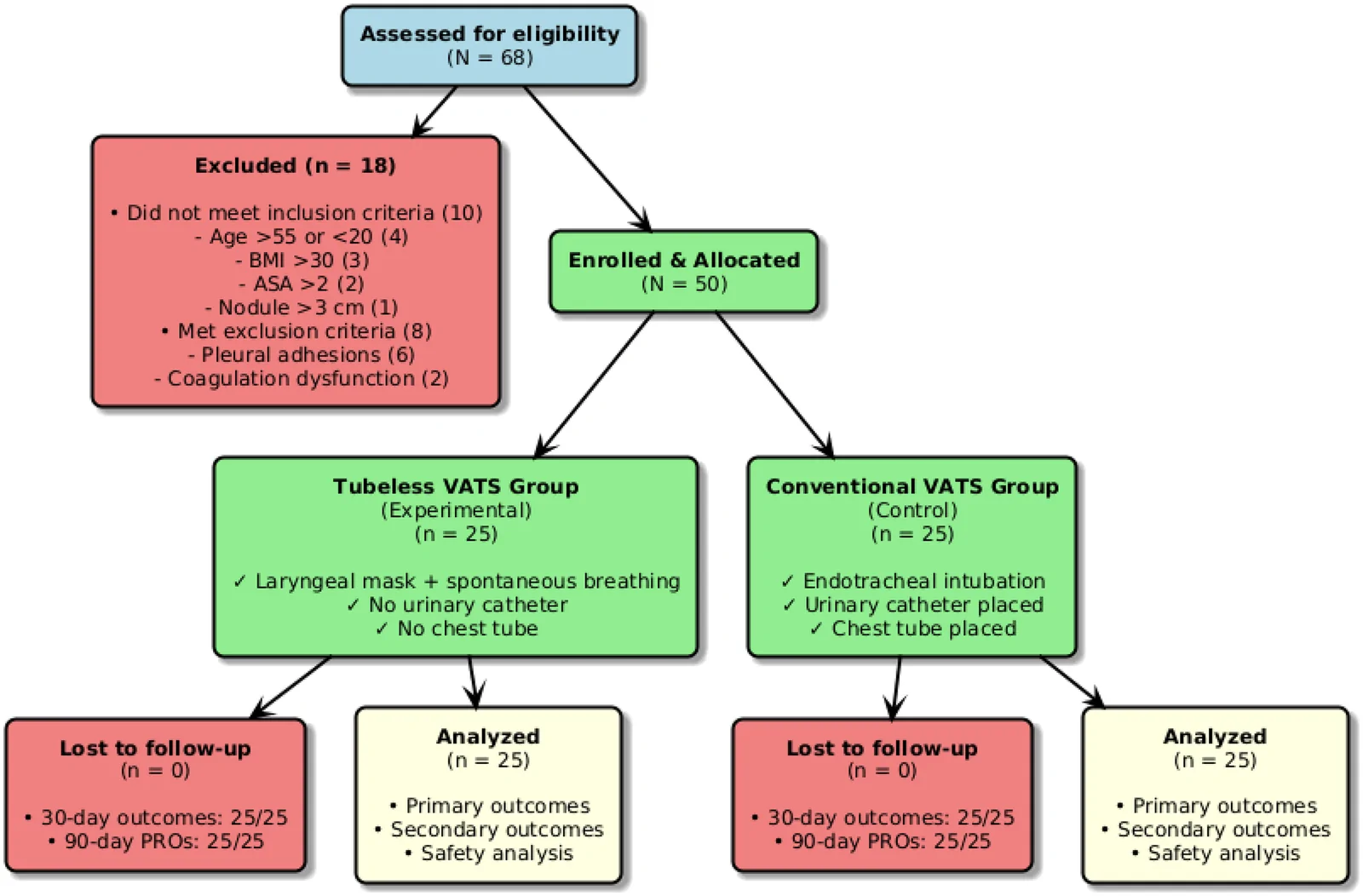
Patient flow diagram. Screening, allocation, and follow-up of 68 patients, with 50 allocated and analyzed. No losses to follow-up. VATS, video-assisted thoracoscopic surgery.

A total of 68 patients were assessed for eligibility, of whom 18 were excluded (10 did not meet the inclusion criteria, and eight met the exclusion criteria). The remaining 50 patients met all inclusion criteria and were enrolled and allocated to either the Tubeless group (*n* = 25) or the conventional VATS group (*n* = 25), as illustrated in Figure 1. All 50 patients successfully underwent VATS without conversion to open surgery. No patient in the Tubeless group required intraoperative conversion to endotracheal intubation or postoperative reoperation. No patients were lost to follow-up.

As shown in Table 1, there were no statistically significant differences between the two groups in age, gender, smoking history, BMI, ASA classification, nodule diameter, pathological type, number of lymph nodes sampled, nodule location, surgical method, or comorbidities (*P* > 0.05 for all).

**Table 1:** Baseline characteristics of patients in both groups.

Characteristic	Tubeless group (*n* = 25)	Conventional group (*n* = 25)	*P*-value
Age (years), mean ± SD	37.24 ± 7.30	37.60 ± 7.90	0.868
Gender, *n* (%)			0.777
Male	13 (52)	14 (56)	
Female	12 (48)	11 (44)	
Smoking history, *n* (%)			0.508
Yes	5 (20)	8 (32)	
No	20 (80)	17 (68)	
BMI (kg/m^2^), mean ± SD	21.53 ± 3.24	23.01 ± 3.67	0.140
ASA classification, *n* (%)			0.773
I	9 (36)	10 (40)	
II	16 (64)	15 (60)	
Nodule diameter (cm), mean ± SD	1.38 ± 0.41	1.41 ± 0.40	0.785
Pathological type, *n* (%)			0.545
Benign nodule	4 (16)	2 (8)	
Adenocarcinoma *in situ*	2 (8)	3 (12)	
Minimally invasive adenocarcinoma	9 (36)	6 (24)	
Invasive carcinoma	10 (40)	14 (56)	
Pathologic stage (invasive carcinoma), *n* (%)			–
Stage IA	10 (100)	14 (100)	
Histologic Subtype (invasive carcinoma), *n* (%)			–
Acinar	4 (40)	6 (43)	
Papillary	3 (30)	4 (29)	
Lepidic	2 (20)	3 (21)	
Micropapillary	1 (10)	1 (7)	
Margin distance (cm), mean ± SD	2.10 ± 0.35	2.05 ± 0.40	0.720
Negative margins, *n* (%)	10 (100)	14 (100)	–
Lymph nodes sampled, n, mean ± SD	3.00 ± 1.70	3.40 ± 1.55	0.426
Nodule location, *n* (%)			0.758
Left upper lobe	7 (28)	6 (24)	
Left lower lobe	4 (16)	6 (24)	
Right upper lobe	7 (28)	8 (32)	
Right middle lobe	1 (4)	2 (8)	
Right lower lobe	6 (24)	3 (12)	
Surgical method, *n* (%)			0.370
Wedge resection	18 (72)	15 (60)	
Segmentectomy	7 (28)	10 (40)	
Comorbidities, *n* (%)			0.464
Yes	3 (12)	6 (24)	
No	22 (88)	19 (76)	

Data are presented as mean ± standard deviation (SD) for continuous variables and as frequency (percentage) for categorical variables. *P*-values were calculated using independent samples *t*-test for continuous variables and chi-square test for categorical variables. Pathologic stage, histologic subtype, margin distance, and negative margins are reported for the subgroup of patients with invasive carcinoma (Tubeless: *n* = 10; Conventional: *n* = 14).

BMI, body mass index; ASA, American Society of Anesthesiologists; SD, standard deviation.

### 3.2 Perioperative clinical indicators

Perioperative clinical indicators are compared in Table 2. There were no statistically significant differences between the two groups in operation time (92.60 ± 22.22 vs. 95.12 ± 18.61 min, *P* = 0.343) or intraoperative blood loss (10.40 ± 4.77 vs. 14.40 ± 9.39 mL, *P* = 0.068). However, the Tubeless group had significantly shorter time to first oral intake (5.12 ± 0.83 vs. 7.68 ± 1.11 h, *P* < 0.001) and significantly shorter postoperative hospital stay (3.00 ± 1.04 vs. 4.84 ± 0.89 days, *P* < 0.001) compared with the conventional group.

**Table 2:** Comparison of perioperative clinical indicators.

Indicator	Tubeless group (*n* = 25)	Conventional group (*n* = 25)	*P*-value
Operation time (min)	92.60 ± 22.22	95.12 ± 18.61	0.343
Intraoperative blood loss (mL)	10.40 ± 4.77	14.40 ± 9.39	0.068
Time to first oral intake (h)	5.12 ± 0.83	7.68 ± 1.11	**<0**.**001**
Postoperative hospital stay (days)	3.00 ± 1.04	4.84 ± 0.89	**<0**.**001**

Data are presented as mean ± standard deviation. *P*-values were calculated using independent samples *t*-test. Statistically significant differences (*P* < 0.05) are shown in bold.

### 3.3 Anesthesia and respiratory indicators

**Figure 2:**
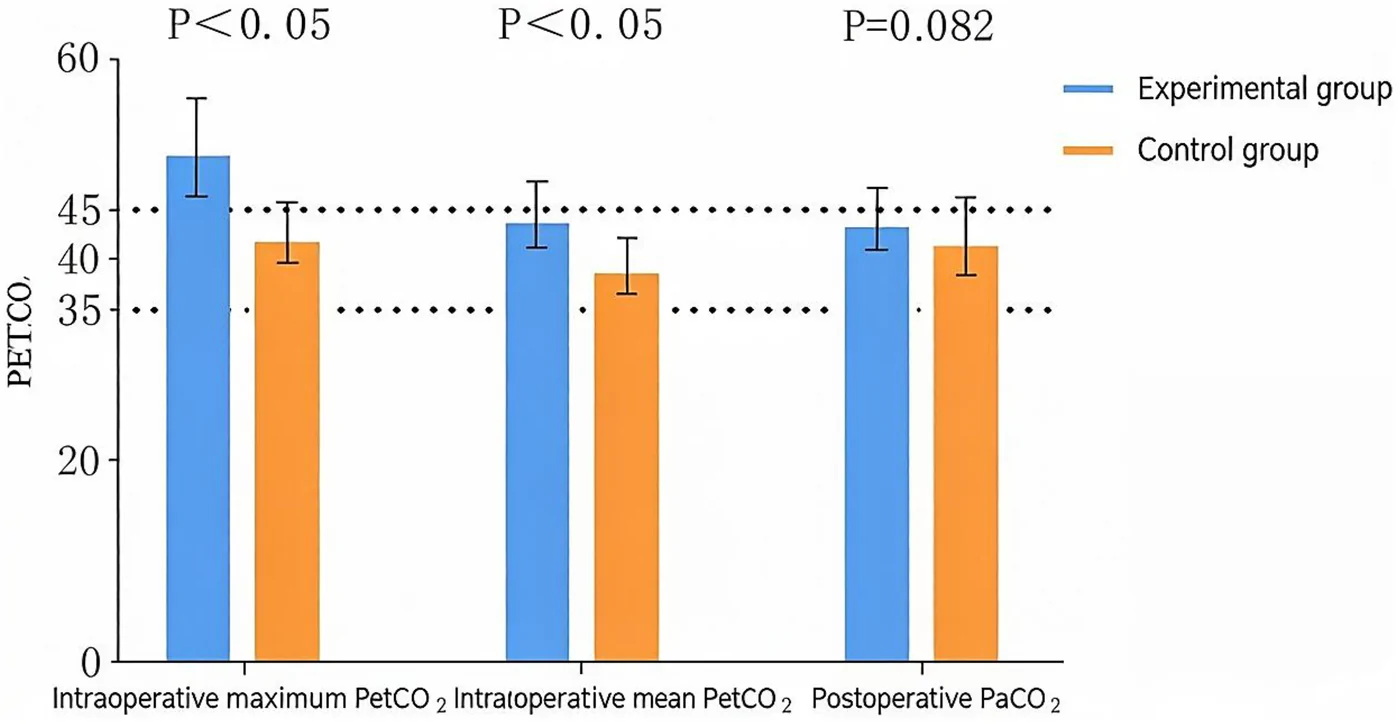
Comparison of anesthesia and respiratory indicators between the two groups. Comparison of intraoperative maximum PetCO_2_, intraoperative mean PetCO_2_, and postoperative PaCO_2_ between the Tubeless group and the conventional group. The Tubeless group showed significantly higher intraoperative maximum PetCO_2_ and intraoperative mean PetCO_2_ than the conventional group, whereas no significant difference was observed in postoperative PaCO_2_. Data are presented as mean ± SD.

Anesthesia and respiratory-related indicators are presented in Table 3 and illustrated in Figure 2. The Tubeless group had significantly higher maximum intraoperative PetCO_2_ (51.20 ± 4.81 vs. 42.64 ± 2.99 mmHg, *P* = 0.023) and mean intraoperative PetCO_2_ (44.52 ± 3.28 vs. 39.44 ± 2.89 mmHg, *P* = 0.031) compared with the conventional group. Anesthesia induction time (15.26 ± 2.53 vs. 20.42 ± 3.65 min, *P* = 0.018) and anesthesia recovery time (11.12 ± 5.74 vs. 23.80 ± 5.82 min, *P* = 0.027) were significantly shorter in the Tubeless group. No significant differences were observed in minimum intraoperative SaO_2_ (93.56 ± 3.47 vs. 94.18 ± 3.82%, *P* = 0.550) or postoperative PaCO_2_ (44.08 ± 3.13 vs. 42.28 ± 3.97 mmHg, *P* = 0.082). No patient in the Tubeless group experienced hemodynamic instability requiring vasopressor support. Assisted ventilation was briefly applied in two patients (8%) in the Tubeless group for transient hypoxemia, which resolved with increased FiO_2_ and jaw-thrust maneuver without requiring conversion to endotracheal intubation.

**Table 3:** Comparison of anesthesia and respiratory indicators.

Indicator	Tubeless group (*n* = 25)	Conventional group (*n* = 25)	*P*-value
Maximum intraoperative PetCO_2_ (mmHg)	51.20 ± 4.81	42.64 ± 2.99	**<0**.**023**
Mean intraoperative PetCO₂ (mmHg)	44.52 ± 3.28	39.44 ± 2.89	**<0**.**031**
Minimum intraoperative SaO_2_ (%)	93.56 ± 3.47	94.18 ± 3.82	0.550
Anesthesia induction time (min)	15.26 ± 2.53	20.42 ± 3.65	**<0**.**018**
Anesthesia recovery time (min)	11.12 ± 5.74	23.80 ± 5.82	**<0**.**027**
Postoperative PaCO₂ (mmHg)	44.08 ± 3.13	42.28 ± 3.97	0.082

Data are presented as mean ± standard deviation. PetCO_2_, end-tidal carbon dioxide partial pressure; SaO_2_, oxygen saturation; PaCO_2_, arterial carbon dioxide partial pressure. *P*-values were calculated using independent samples *t*-test.

Bold values indicate statistically significant differences (*P* < 0.05).

### 3.4 Postoperative inflammatory indicators

Postoperative inflammatory markers measured on postoperative day 1 are summarized in Table 4. The Tubeless group had significantly lower white blood cell count (10.67 ± 1.13 vs. 11.75 ± 1.62 × 10⁹/L, *P* = 0.009) and significantly lower NLR (8.23 ± 2.48 vs. 9.85 ± 3.12, *P* = 0.048) compared with the conventional group. No significant differences were observed in platelet count (*P* = 0.498), neutrophil count (*P* = 0.290), lymphocyte count (*P* = 0.440), PLR (*P* = 0.170), or SII (*P* = 0.140).

**Table 4:** Comparison of postoperative inflammatory indicators (POD 1).

Indicator	Tubeless group (*n* = 25)	Conventional group (*n* = 25)	*P*-value
WBC count (× 10⁹/L)	10.67 ± 1.13	11.75 ± 1.62	**0**.**009**
Platelet count (× 10⁹/L)	204.63 ± 33.50	197.85 ± 36.73	0.498
Neutrophil count (× 10⁹/L)	9.25 ± 1.74	9.81 ± 1.94	0.290
Lymphocyte count (× 10⁹/L)	1.11 ± 0.38	1.04 ± 0.24	0.440
NLR	8.23 ± 2.48	9.85 ± 3.12	**0**.**048**
PLR	182.37 ± 23.51	192.84 ± 29.36	0.170
SII	1,678.50 ± 632.51	1,930.43 ± 564.67	0.140

Data are presented as mean ± standard deviation. POD, postoperative day; WBC, white blood cell; NLR, neutrophil-to-lymphocyte ratio; PLR, platelet-to-lymphocyte ratio; SII, systemic immune-inflammation index (calculated as platelet count   ×   neutrophil count/lymphocyte count). *P*-values were calculated using independent samples *t*-test. Statistically significant differences (*P* < 0.05) are shown in bold.

### 3.5 Postoperative complications

Postoperative complications graded by the Clavien–Dindo classification are summarized in Table 5. The Tubeless group had significantly fewer patients with complications [2 [8%] vs. 8 [32%], *P* = 0.034]. However, it should be noted that the complications in the conventional group (sore throat, hoarseness, urinary tract infection) were predominantly related to intubation and catheterization, whereas the Tubeless group’s complications were directly related to the surgical procedure itself (pneumothorax). In the Tubeless group, two patients developed pneumothorax on postoperative chest X-ray: One required closed thoracic drainage (Grade IIIa, representing a failure of the complete Tubeless strategy), and one had minimal pneumothorax that resolved spontaneously (Grade I). In the conventional group, all complications were Grade I and included sore throat (*n* = 5), subcutaneous emphysema (*n* = 1), transient hoarseness (*n* = 1), and urinary tract infection (*n* = 1).

**Table 5:** Comparison of postoperative complications graded by Clavien–Dindo classification.

Complication	Tubeless group (*n* = 25)	Conventional group (*n* = 25)	Clavien-Dindo grade	*P*-value
Any complication, *n* (%)	2 (8)	8 (32)	–	**0.034**
Grade I				
Sore throat	0	5	I	
Hoarseness	0	1	I	
Subcutaneous emphysema	0	1	I	
Urinary tract infection	0	1	I	
Pneumothorax (resolved spontaneously)	1	0	I	
Grade IIIa				
Pneumothorax requiring drainage	1	0	IIIa	

Clavien–Dindo classification: Grade I, deviation from normal postoperative course without need for pharmacological or interventional treatment; Grade II, requiring pharmacological treatment; Grade IIIa, requiring intervention without general anesthesia. Conventional group complications (sore throat, hoarseness, urinary tract infection) were predominantly related to intubation and catheterization, whereas Tubeless group complications (pneumothorax) were directly related to the surgical procedure itself. *P*-value for any complication was calculated using Fisher's exact test.

Bold values indicate statistically significant differences (*P* < 0.05).

### 3.6 VAS pain scores

**Figure 3:**
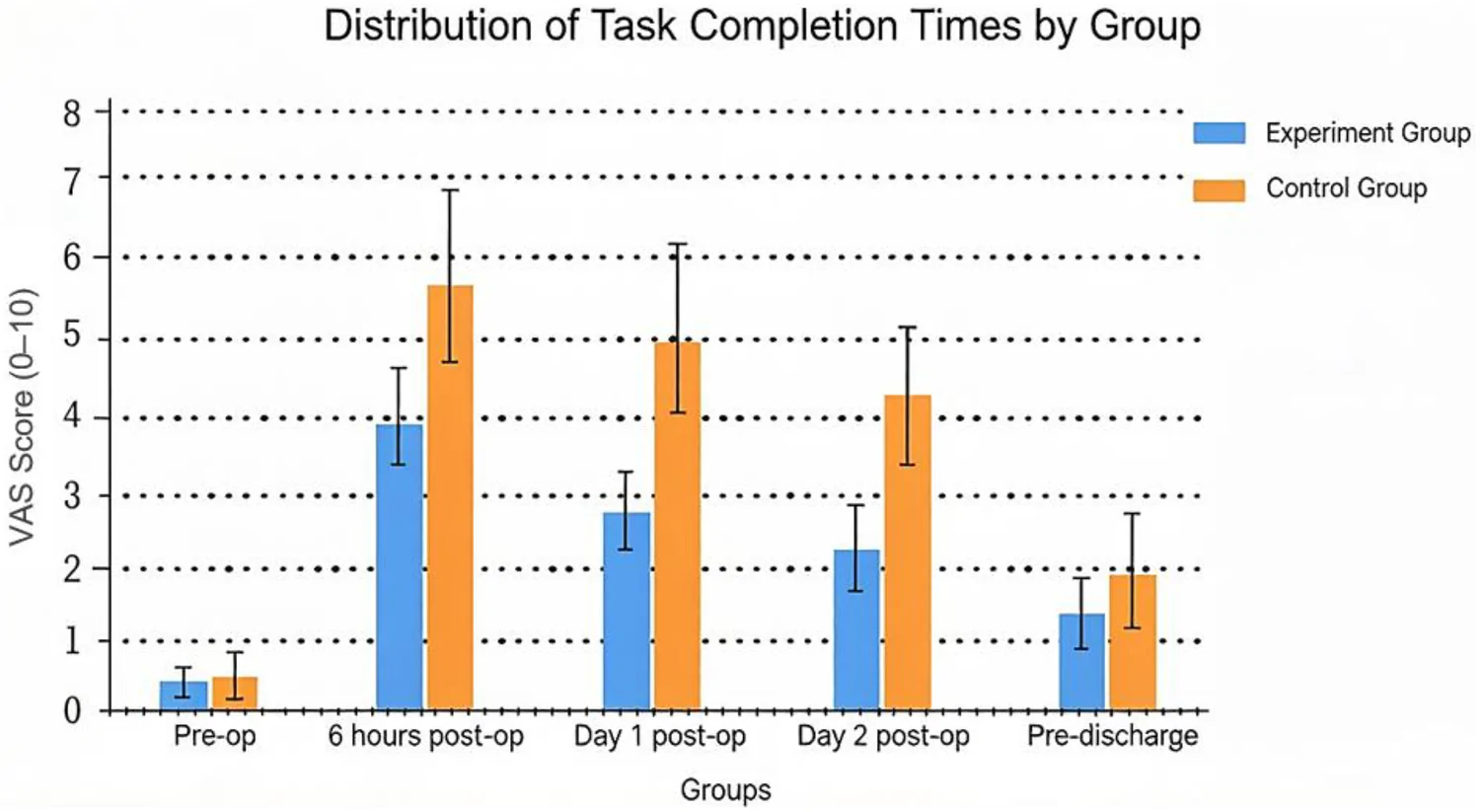
Comparison of VAS pain scores between the two groups. Comparison of VAS pain scores between the Tubeless group and the conventional group at the preoperative stage, 6 h postoperatively, postoperative day 1, postoperative day 2, and before discharge. The Tubeless group had significantly lower VAS pain scores at all postoperative time points, whereas no significant difference was observed preoperatively. Data are presented as mean ± SD. VAS, visual analog scale (0 = no pain, 10 = worst possible pain).

VAS pain scores are presented in Table 6 and illustrated in Figure 3. Preoperative VAS scores did not differ between groups (0.88 ± 0.44 vs. 0.84 ± 0.56, *P* = 0.779). The Tubeless group had significantly lower VAS scores at 6 h postoperatively (3.88 ± 0.66 vs. 5.64 ± 1.22, *P* < 0.01), on postoperative day 1 (2.75 ± 0.53 vs. 4.96 ± 1.17, *P* < 0.01), postoperative day 2 (2.23 ± 0.56 vs. 4.17 ± 0.95, *P* < 0.01), and before discharge (1.36 ± 0.49 vs. 1.93 ± 0.78, *P* < 0.01).

**Table 6:** Comparison of VAS pain scores.

Time point	Tubeless group (*n* = 25)	Conventional group (*n* = 25)	*P*-value
Preoperative	0.88 ± 0.44	0.84 ± 0.56	0.779
6 h postoperatively	3.88 ± 0.66	5.64 ± 1.22	**<0**.**01**
Postoperative day 1	2.75 ± 0.53	4.96 ± 1.17	**<0**.**01**
Postoperative day 2	2.23 ± 0.56	4.17 ± 0.95	**<0**.**01**
Before discharge	1.36 ± 0.49	1.93 ± 0.78	**<0**.**01**

Data are presented as mean ± standard deviation. VAS = visual analog scale (0 = no pain, 10 = worst possible pain). *P*-values were calculated using independent samples *t*-test.

Bold values indicate statistically significant differences (*P* < 0.05).

### 3.7 Patient-Reported outcomes (MDASI scores)

**Figure 4:**
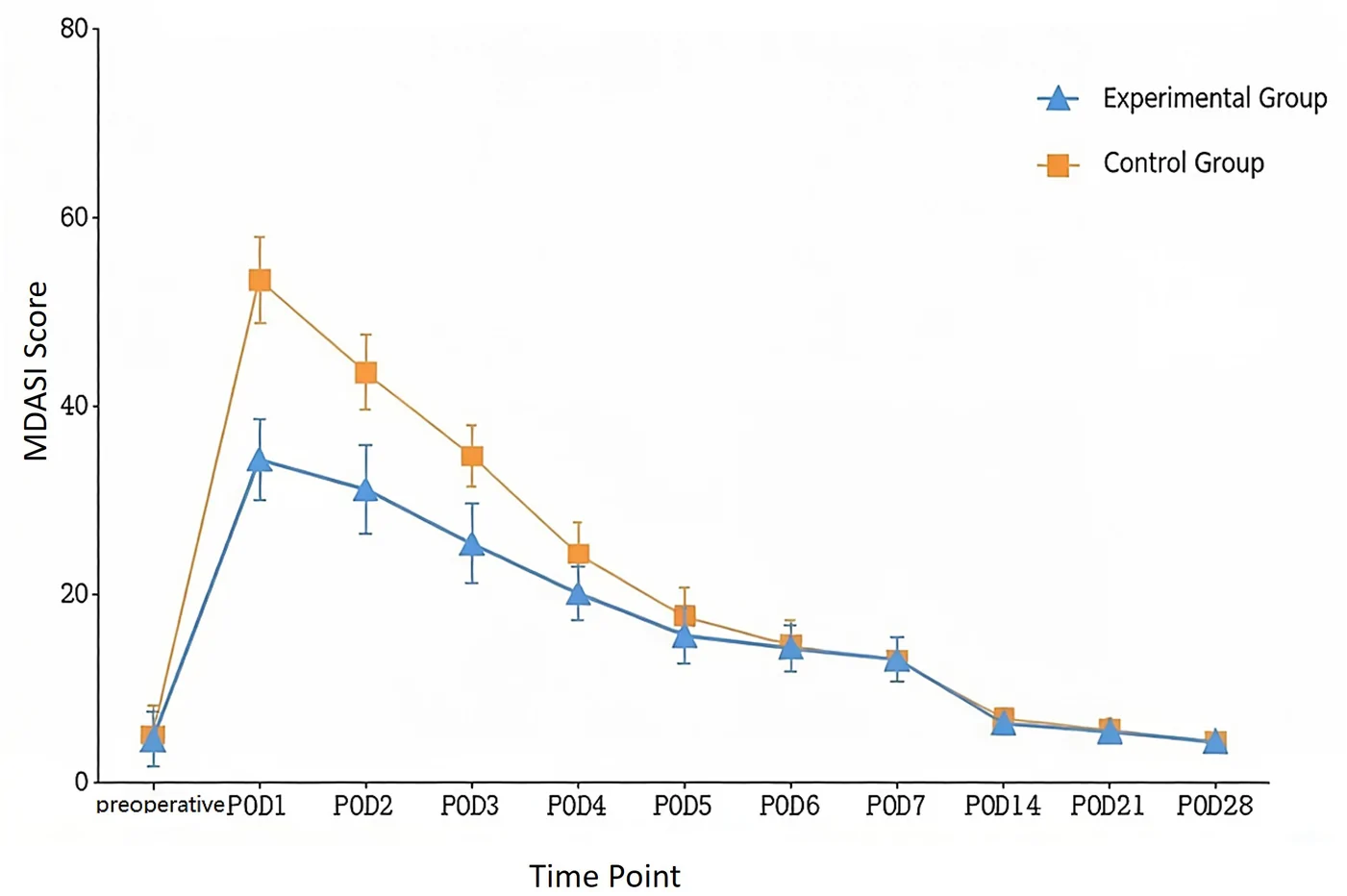
Comparison of patient-reported outcomes (MDASI scores) between the two groups. MDASI scores were compared from preoperative to postoperative week 4. The Tubeless group showed significantly lower symptom burden from postoperative day 1 through day 5 (*P* < 0.05 for all). The between-group difference gradually diminished over time, with both groups returning to similar symptom levels by postoperative day 6. Data are presented as mean ± SD. MDASI, MD Anderson Symptom Inventory.

MDASI scores are presented in Table 7 and illustrated in Figure 4. The Tubeless group had significantly lower MDASI scores from postoperative day 1 through day 5 (*P* < 0.05 for all). In particular, on postoperative day 1, MDASI scores were 34.24 ± 4.31 in the Tubeless group versus 53.28 ± 4.60 (*P* < 0.001) in the conventional group; on day 2, 31.04 ± 4.65 vs. 43.48 ± 3.93 (*P* < 0.001); on day 3, 25.32 ± 4.10 vs. 34.64 ± 3.26 (*P* < 0.001); on day 4, 20.54 ± 2.87 vs. 24.26 ± 3.20 (*P* < 0.001); and on day 5, 15.60 ± 2.94 vs. 17.56 ± 2.95 (*P* = 0.039). The difference between groups was most pronounced during the early postoperative period, with both groups returning to similar symptom levels by the end of the second postoperative week. No significant differences were observed preoperatively (*P* = 0.639), on postoperative days 6–7 (*P* = 0.617 and *P* = 0.905, respectively), or at postoperative weeks 2, 3, and 4 (*P* = 0.318, *P* = 0.440, and *P* = 0.614, respectively).

**Table 7:** Comparison of patient-reported outcomes (MDASI scores).

Time point	Tubeless group (*n* = 25)	Conventional group (*n* = 25)	*P*-value
Preoperative	4.48 ± 2.85	5.00 ± 3.30	0.639
Postoperative day 1	34.24 ± 4.31	53.28 ± 4.60	**<0**.**001**
Postoperative day 2	31.04 ± 4.65	43.48 ± 3.93	**<0**.**001**
Postoperative day 3	25.32 ± 4.10	34.64 ± 3.26	**<0**.**001**
Postoperative day 4	20.54 ± 2.87	24.26 ± 3.20	**<0**.**001**
Postoperative day 5	15.60 ± 2.94	17.56 ± 2.95	**0**.**039**
Postoperative day 6	14.16 ± 2.35	14.52 ± 2.69	0.617
Postoperative day 7	13.04 ± 2.22	12.96 ± 2.49	0.905
Postoperative week 2	6.28 ± 1.42	6.72 ± 1.17	0.318
Postoperative week 3	5.32 ± 1.06	5.58 ± 1.29	0.440
Postoperative week 4	4.28 ± 0.86	4.40 ± 0.81	0.614

Data are presented as mean ± standard deviation. MDASI = MD Anderson Symptom Inventory (core items: pain, fatigue, sleep disturbance, shortness of breath, coughing, appetite loss, constipation; each item scored 0–10, with higher scores indicating worse symptoms). *P*-values were calculated using independent samples *t*-test. Statistically significant differences (*P* < 0.05) are shown in bold.

## 4 Discussion

This study compared Tubeless VATS (spontaneous breathing with laryngeal mask anesthesia, without urinary catheter or chest tube) versus conventional intubated VATS in 50 young and middle-aged patients undergoing wedge or segmental resection for peripheral pulmonary nodules ≤3 cm. Our findings suggest that Tubeless VATS appears safe and feasible in this selected cohort, with no intraoperative conversions to intubation, no reoperations, and no losses to follow-up. Compared with conventional VATS, the Tubeless group demonstrated significantly shorter time to first oral intake (5.12 vs. 7.68 h), shorter postoperative hospital stay (3.00 vs. 4.84 days), lower VAS pain scores at all postoperative time points, and fewer overall complications (8% vs. 32%),with conventional group complications being predominantly minor Grade I events related to intubation and catheterization. We acknowledge that the Tubeless intervention comprises multiple simultaneous components, including airway strategy, ventilation, urinary catheter avoidance, chest tube avoidance, and regional analgesia. Therefore, the observed reductions in pain and hospital stay cannot be attributed solely to any single component of the Tubeless approach, and the avoidance of chest tube alone may explain much of the analgesic benefit. This limitation should be considered when interpreting our findings. In addition, the Tubeless group demonstrated reduced postoperative inflammatory response, evidenced by significantly lower white blood cell count and NLR on postoperative day 1. Patient-reported outcomes (MDASI scores) were also significantly better in the Tubeless group from postoperative day 1 through day 5, although this benefit was not sustained beyond day 6. These findings are consistent with the principles of ERAS and suggest that Tubeless VATS may offer advantages in selected patients.

The safety of non-intubated spontaneous breathing anesthesia in thoracic surgery has been well established in the literature. The first demonstration of awake wedge resection under epidural anesthesia was reported nearly two decades ago (8). Since then, the non-intubated approach has been extended to more complex procedures, including uniportal VATS lobectomy and systematic lymph node dissection (9). A large systematic review and meta-analysis encompassing 1,684 cases confirmed that non-intubated VATS is safe, effective, and feasible across a wide range of thoracic diseases (10). In the setting of primary palmar hyperhidrosis, spontaneous ventilation anesthesia combined with uniportal Tubeless thoracoscopic sympathectomy achieved an average hospital stay of only 1 day in 144 patients (11). For primary spontaneous pneumothorax, non-intubated VATS resulted in shorter anesthesia recovery time, shorter postoperative hospital stay, lower pain scores, reduced hospitalization costs, and lower incidence of postoperative sore throat compared with intubated VATS (12). Tubeless VATS has also been successfully applied to mediastinal tumor resection in 67 patients with zero postoperative complications at discharge (13). Our study extends these findings by specifically focusing on young and middle-aged patients with low comorbidity burden undergoing sublobar resections, and by incorporating both objective inflammatory markers (NLR, PLR, SII) and patient-reported outcomes (MDASI) as endpoints.

With regard to anesthesia indicators, we observed significantly higher intraoperative PetCO_2_ in the Tubeless group. This finding can be explained by the fact that air entering the pleural cavity creates an iatrogenic pneumothorax, increasing intrathoracic pressure on the affected side, compressing lung tissue, and potentially causing mediastinal swing, which collectively reduce CO_2_ elimination (14). Importantly, the primary reason for intraoperative conversion to intubation is hypoxemia, and mild hypercapnia does not lead to hemodynamic instability or increase the risk of intubation (5). In our study, postoperative PaCO_2_ returned to normal rapidly, suggesting that transient hypercapnia is well tolerated. Furthermore, spontaneous breathing and diaphragmatic movement generate negative intrathoracic pressure, which promotes blood return and maintains intraoperative hemodynamic stability, potentially resulting in better lung perfusion and ventilation compared with one-lung ventilation, with a lower risk of perioperative hypoxemia (15). Most cases of intraoperative hypoxemia can be managed with assisted ventilation and increasing oxygen concentration.

The low rate of assisted ventilation (8%) and absence of hemodynamic instability in the Tubeless group suggest that respiratory and hemodynamic complications are uncommon with this approach. However, PetCO_2_ monitoring during spontaneous ventilation and open pneumothorax conditions should be interpreted with caution, as it may not accurately reflect PaCO_2_ due to ventilation–perfusion mismatch.

With respect to surgical outcomes, our study found no significant differences between groups in operation time, intraoperative blood loss, or number of lymph nodes sampled. These findings are consistent with previous reports indicating that non-intubated anesthesia does not compromise surgical progression or lymph node dissection (16, 17). The traditional view holds that systematic mediastinal lymph node dissection is important for radical lung cancer surgery; however, for early-stage lung cancer, clinicians increasingly favor lymph node sampling over complete dissection. Our results suggest that Tubeless technology does not appear to adversely affect the ability to perform lymph node sampling, although the limited number of sampled nodes (mean 3.0–3.4) precludes definitive conclusions about oncologic adequacy.

The postoperative inflammatory response was attenuated in the Tubeless group, with lower white blood cell counts and lower NLR compared with the conventional group. These findings are consistent with a previous study comparing non-intubated versus intubated VATS lobectomy, which also reported reduced inflammatory markers in the non-intubated group (18). This attenuated inflammatory response may indicate that Tubeless VATS is associated with less surgical trauma, which could contribute to faster postoperative recovery. With regard to rehabilitation indicators, the Tubeless group had significantly shorter time to first oral intake and shorter postoperative hospital stay. Reduced use of intravenous and inhalational anesthetics may minimize the impact on gastrointestinal nerve function (7). In addition, avoiding chest tubes may reduce postoperative pain and enable earlier ambulation (19). Our VAS pain scores showed that the Tubeless group experienced less pain at all postoperative time points.

The observational design and exploratory nature of this study mean that our findings should be interpreted with caution. While the observed differences were statistically significant, the absence of randomization and the small sample size preclude definitive conclusions regarding superiority. Therefore, these results should be considered hypothesis-generating and warrant confirmation in future prospective, randomized, and adequately powered studies.

### 4.1 Limitations and future research

This study has several limitations. First, it was a single-center, retrospective, non-randomized design, which introduces potential selection bias despite comparable baseline characteristics. Allocation was not randomized; instead, patients were assigned to groups based on clinical decision-making and patient preference. The surgical team's experience and perioperative protocols may also limit generalizability. Second, the sample size (*n* = 50) was relatively small, and we did not perform an *a priori* sample size calculation. Third, we performed multiple unadjusted comparisons across several outcome measures, increasing the risk of Type I error; therefore, our findings should be interpreted as exploratory and hypothesis-generating rather than confirmatory. Fourth, our inclusion criteria were restrictive (age 20–55 years, BMI ≤30 kg/m^2^, ASA ≤2), limiting generalizability to older, frailer, or higher-risk patients. Patients undergoing lobectomy were excluded, further narrowing generalizability. Fifth, we did not assess long-term oncologic outcomes such as recurrence-free or overall survival in patients with invasive carcinoma (*n* = 24), leaving unanswered whether Tubeless anesthesia affects long-term outcomes. Sixth, maximum PaCO_2_ values, duration of hypercapnia, and detailed hypoxemia episode data were not systematically recorded in our retrospective dataset, limiting the depth of our respiratory safety analysis. Seventh, the modified 7-item MDASI used in our study has not been formally validated for this specific context, and repeated measures were analyzed using multiple independent *t*-tests rather than a longitudinal mixed-effects model, which may increase the risk of Type I error. Eighth, the limited number of lymph nodes sampled (mean 3.0–3.4 nodes per group) and the absence of long-term recurrence or survival data preclude conclusions about oncologic adequacy. Our findings should be interpreted as short-term feasibility and recovery outcomes only.

Future multicenter prospective randomized controlled trials with larger sample sizes and propensity score matching are needed to confirm our findings. Such studies should include patients undergoing lobectomy, extend follow-up to 3–5 years for oncologic outcomes, and validate a thoracic-specific patient-reported outcome measure.

## 5 Conclusion

In selected young and middle-aged patients with peripheral pulmonary nodules ≤3 cm undergoing wedge or segmental resection, complete Tubeless VATS appears to be a safe and feasible alternative to conventional intubated VATS. In this cohort, Tubeless VATS was associated with reduced postoperative pain, shorter hospital stay, lower inflammatory response, fewer complications (particularly minor airway and urinary complications related to intubation and catheterization), and improved early patient-reported outcomes. This Tubeless technique is consistent with ERAS principles and may warrant consideration as an alternative approach in this specific patient population. However, our findings are limited to short-term outcomes, and further prospective multicenter studies with long-term oncologic follow-up are needed to confirm these findings and assess oncologic adequacy.

## Data Availability

The original contributions presented in the study are included in the article/Supplementary Material, further inquiries can be directed to the corresponding authors.
